# Fortified Cereal-Based Foodstuffs: Technological, Sensory, and Nutritional Properties

**DOI:** 10.3390/foods13081182

**Published:** 2024-04-12

**Authors:** Roberta Tolve, Barbara Simonato

**Affiliations:** Department of Biotechnology, University of Verona, Strada Le Grazie 15, 37134 Verona, Italy

In the wake of the United Nations’ Agenda 2030, a global commitment to advancing well-being, sustainable living, and waste reduction, the spotlight on cereal-based food products with high added value has intensified. The escalating demand for healthful products positively impacting human nutrition has paved the way for innovative food science and technology solutions [1]. Fortification, a method of incorporating bioactive compounds into everyday foods, has emerged as a pivotal strategy to meet consumer needs while enhancing foodstuffs’ nutritional and health properties [2]. Cereal-based products, serving as the bedrock of global nourishment, present a fertile ground for fortification with essential elements such as fiber, phytochemicals, protein, fatty acids, vitamins, and minerals [3]. Additionally, within the circular economy framework, there is a call to harness the potential of agro-industrial by-products endowed with high added value to pursue sustainable and efficient food systems [4]. The waste generated from the food supply chain, including agro-industrial by-products, accounts for around 30% of the total food produced globally. Fortifying cereal-based products, which are fundamental sources of nourishment, could aid waste reduction on a global scale [5].

Considering the possibility of fortifying food products with by-products from the food processing industry, Lomuscio et al. [6] delve into the potential of utilizing brewing by-products, particularly trub, which are rich in proteins and fibers, to enhance the nutritional profile of durum wheat fresh pasta. Their research sheds light on the intricate technological and physical–chemical properties influenced by the incorporation of debittered trub powder. The study underscores the fact that debittered trub, at a substitution level of up to 10%, holds promise as an ingredient for fortified fresh pasta, enriching its nutritional content without compromising quality or sensory attributes. Trub is among the three primary wastes generated in the brewing industry, alongside brewers’ spent grain and residual brewers’ yeast. Both of these by-products and derivatives thereof have been utilized to fortify cereal-based products. For instance, research has demonstrated that adding brewers’ spent grain protein hydrolysates, which possess diverse biological properties, including antioxidant activity, into muffin formulations has promising antidiabetic properties [7]. Moreover, recent research has evaluated the possibility of using β-glucan from brewers’ spent yeast as a techno-functional food ingredient for pasta fortification [8].

In recent years, many ingredients have been used to fortify various cereal-based products. Generally, cereal-based products that are globally accepted by consumers are selected and taken in such a way as to convey the bioactive compounds contained in the fortifiers. For instance, among bakery items, pizza is consumed and enjoyed worldwide. Due to the simplicity of its preparation and good taste, pizza is also a popular snack that could be a promising vehicle for functional compounds, thus satisfying health-conscious customers [9,10]. Over the years, this beloved comfort food has seen fortification with by-products from cauliflower processing [11] and soybean oil and carrot extract [12]. More recently, the exploration has expanded to developing a functional pizza base enriched with jujube powder, harnessing the health advantages of this distinctive ingredient. The study delves into chemical analyses, unveiling heightened phenolic compounds and antioxidant activity. This research demonstrates how jujube powder can be effectively integrated into pizza dough, introducing a fresh dimension to this cherished comfort food [13].

Similarly, Asian noodles, including udon, ramen, Korean white salted noodles, and Chinese yellow alkaline noodles, are widely enjoyed. The rheological properties of salted noodles are closely tied to the composition of the wheat flour used. With consumers increasingly seeking foods with added health benefits, incorporating composite flours rich in resistant starch (RS) could be crucial for producing nutritious noodles. There is a growing tendency to boost dietary fiber, such as RS, in essential ingredients like wheat flour for noodle preparation, aiming to provide extra health benefits during digestion [14]. In salted noodles, incorporating highly resistant starches, such as high-amylose corn starch, heat moisture treatment corn starch, and green banana flour, takes center stage. The study unravels the intricate relationship between these alternative flours and the physical properties of dough, impacting the eating quality and estimated glycemic index of salted noodles. The findings suggest that such incorporation can lead to the development of low-glycemic noodles with good acceptability, contributing to gastrointestinal health [15]. This Special Issue also delves into fortifying standard wheat-based crackers with faba-bean-derived components, revealing improved macronutrient composition and increased dietary fiber, fat, and resistant starch. This exploration showcases the potential of faba bean flour, starch concentrate, protein concentrate, and protein isolate to enhance wheat-based crackers’ nutritional properties and functional value [16].

Additionally, this Editorial includes a paper addressing the current lifestyle trends and the growing consumer interest in healthier bread products with improved nutritional compositions. The paper explores the incorporation of various protein sources, both animal and vegetable. It analyzes the impact on nutritional changes, dough properties, texture parameters, appearance, flavor, and health-related effects. Alternative processing biotechnologies, such as germination, fermentation, and sourdough-based methods, are discussed, emphasizing their potential to enhance the dough’s composition and nutritional properties. For example, it has been reported that using sourdough and germinated flour improves the rate of starch hydrolysis related to the glycemic index. These alternative processing biotechnologies are highlighted as a strategy to positively impact bread’s texture, appearance, flavor, and aroma, ultimately contributing to consumer health.

This underscores the importance of innovating alternative protein sources and implementing technological strategies for their better incorporation, ensuring the maintenance of texture and enhancement of sensory properties in bread products [17].

## Conclusions

In conclusion, the research articles on fortified cereal-based foodstuffs presented in this Special Issue reflect a food development revolution, emphasizing their pivotal role in shaping a future where nutrition, sustainability, and palatability coexist. These innovations underscore significant strides in fortification, contributing to a healthier, more sustainable world. The diverse approaches showcased in this collection align with the global commitment outlined in the United Nations’ Agenda 2030, highlighting the transformative potential of fortified cereals to address pressing challenges. Together, they pave the way for a future where fortified cereals are central to addressing global concerns and fostering positive change.
